# Discovery of Compounds that Positively Modulate the High Affinity Choline Transporter

**DOI:** 10.3389/fnmol.2017.00040

**Published:** 2017-02-27

**Authors:** Parul Choudhary, Emma J. Armstrong, Csilla C. Jorgensen, Mary Piotrowski, Maria Barthmes, Rubben Torella, Sarah E. Johnston, Yuya Maruyama, John S. Janiszewski, R. Ian Storer, Sarah E. Skerratt, Caroline L. Benn

**Affiliations:** ^1^PfizerNeusentis, Cambridge, UK; ^2^Primary Pharmacology Group, Pfizer Inc.Groton, CT, USA; ^3^Nanion TechnologiesMunich, Germany; ^4^Pfizer, Worldwide Medicinal ChemistryCambridge, UK; ^5^Central Research Laboratory, Kissei Pharmaceutical Co., Ltd.Nagano, Japan

**Keywords:** HACU (high affinity choline uptake), acetylcholine, solute carrier, SSM electrophysiology, phenotypic screening, mass spectrometry, small molecule screening, SLC5A7

## Abstract

Cholinergic hypofunction is associated with decreased attention and cognitive deficits in the central nervous system in addition to compromised motor function. Consequently, stimulation of cholinergic neurotransmission is a rational therapeutic approach for the potential treatment of a variety of neurological conditions. High affinity choline uptake (HACU) into acetylcholine (ACh)-synthesizing neurons is critically mediated by the sodium- and pH-dependent high-affinity choline transporter (CHT, encoded by the *SLC5A7* gene). This transporter is comparatively well-characterized but otherwise unexplored as a potential drug target. We therefore sought to identify small molecules that would enable testing of the hypothesis that positive modulation of CHT mediated transport would enhance activity-dependent cholinergic signaling. We utilized existing and novel screening techniques for their ability to reveal both positive and negative modulation of CHT using literature tools. A screening campaign was initiated with a bespoke compound library comprising both the Pfizer Chemogenomic Library (CGL) of 2,753 molecules designed specifically to help enable the elucidation of new mechanisms in phenotypic screens and 887 compounds from a virtual screening campaign to select molecules with field-based similarities to reported negative and positive allosteric modulators. We identified a number of previously unknown active and structurally distinct molecules that could be used as tools to further explore CHT biology or as a starting point for further medicinal chemistry.

## Introduction

Cholinergic neurons are responsible for transmitting signals to a wide range of tissues within the peripheral and central nervous systems. As a result, they are involved in a variety of crucial biological processes including muscle contraction, cognition, learning, memory, and control of autonomic functions (Woolf and Butcher, 2011). Decreased acetylcholine (ACh) levels or expression and/or function of the neurotransmitter receptors, in selected areas of the nervous system, have been described in several neurodegenerative diseases such as Alzheimer's, Parkinson's and Huntington's as well as in psychiatric disorders such as schizophrenia. The high affinity choline transporter (CHT) is responsible for uptake of choline into cholinergic nerve terminals, where it is acetylated by choline acetyltransferase (ChAT) to form the neurotransmitter acetylcholine. The transporter protein was cloned and identified by Okuda and co-workers in 2000, and shown to exhibit high-affinity, sodium-dependent choline uptake (*K*_*m*_~2 μM) which could be inhibited by hemicholinium-3 (HC-3) with a *K*_*i*_ of 1–5 nM (Okuda and Haga, 2000; Okuda et al., 2000; Apparsundaram et al., 2001). Collective evidence indicates that CHT density in the synaptic plasma membrane is the primary variable determining the capacity of cholinergic neurotransmission (Ferguson and Blakely, 2004; Ribeiro et al., 2006; Black and Rylett, 2012). The majority of CHT is localized intracellularly including a proportion present on ACh-containing synaptic vesicles, suggesting an elegant mechanism for linking ACh release to CHT membrane density and choline re-uptake (Ferguson et al., 2003; Apparsundaram et al., 2005): vesicular fusion is able to support a rapid biosynthetic response to neuronal stimulation. Manipulations that increase the rate of choline uptake, *V*_*max*_, also increase transporter density in the synaptic membrane. Post-translational modifications (PTM) such as phosphorylation and ubiquitination have been shown to modulate activity state in addition to subcellular trafficking via endosomal compartments into synaptic vesicles (Cooke and Rylett, 1997; Kar et al., 1998; Gates et al., 2004; Misawa et al., 2008; Black et al., 2010; Yamada et al., 2012; Hartnett et al., 2014; Parikh et al., 2014). Additional regulation likely includes specific protein-protein interactions; however the complex partners are not well-defined (Kar et al., 1998; Ribeiro et al., 2003; Parikh et al., 2006, 2014; Brock et al., 2007; Misawa et al., 2008; Pinthong et al., 2008; Cuddy et al., 2012, 2014, 2015; Kristofikova et al., 2013; Fishwick and Rylett, 2015). This raises the possibility of modulating CHT surface localization to impact on transport *V*_*max*_ in addition to direct modulation of transport function through affinity (*K*_*m*_) or rate.

Presynaptic mechanisms influencing ACh synthesis and release have received little attention as therapeutic strategies for modulating cholinergic function, despite good understanding and high conservation of cellular mechanisms. Indeed, ACh release cannot be sustained without presynaptic transporter mediated recapture of choline, as demonstrated by genetic and pharmacological studies (Ferguson et al., 2004; Ferguson and Blakely, 2004; reviewed in Brandon et al., 2004; Apparsundaram et al., 2005; Parikh and Sarter, 2006; Bazalakova et al., 2007; Parikh et al., 2013). Neurons enhance CHT activity in response to neuronal activity to enhance ACh production. Thereby, if this mechanism were to be modulated by compounds, this offers a potentially impactful approach to augment cholinergic signaling for therapeutic purposes. Furthermore, the mechanism focuses on clearance of choline from the synapse rather than ectopic stimulation of acetylcholine receptors through acetylcholinesterase inhibitor (AChEI) treatment.

We therefore sought to leverage the comparatively high level of characterization and potential interest of CHT as a target to develop a platform to assess existing and novel approaches to characterizing electrogenic transporter function in a drug discovery context. We prioritized approaches focused on direct assessment of transporter function as an output that could be developed further into primary medium- and high-throughput screens. We also assessed potential secondary screening modalities using alternative assay formats such as assessment of transporter localization. We performed a focused screening campaign using a bespoke compound set. There is comparative paucity of relevant known tool molecules that modulate CHT function: published orthosteric inhibitors include hemicholinium-3 (HC-3) and related analogs which have been broadly used since their first discovery in 1955 (Ferguson and Blakely, 2004). In addition there is the recently described negative allosteric modulator (NAM) ML-352 (Ennis et al., 2015) and putative positive modulators of transporter function including MKC-231/coluracetam (Bessho et al., 2008; Takashina et al., 2008a,b) and staurosporine (STS) (Ruggiero et al., 2012). ML-352 and MKC-231 were used as seeds for generating the first set of 887 compounds via Cresset software (a computational approach that generates a 3-dimensional electrostatic shape or “field” which illustrates how the compound may interact with the target). The second set comprised the Pfizer Chemogenomic Library (CGL) of 2,753 compounds specifically designed to support target identification from phenotypic screening (Jones and Bunnage, 2017). We identified a number of active, previously unknown CHT positive allosteric modulator (PAM) chemotypes that could be used either as tools or as starting points for further medicinal chemistry activities.

## Materials and methods

### Cell line generation and culture

Sequences corresponding to full-length CHT (NM_021815.4) and LV-AA mutation at residues 531–532 (Ribeiro et al., 2005; Ruggiero et al., 2012) were codon-optimized for expression in human cell lines and custom-synthesized by GeneArt (LifeTech). Constructs were designed with a N-terminal FLAG epitope (Cuddy et al., 2012) and cloned into the pLenti6.3/V5 DEST vector which also contained a C-terminal V5 epitope tag (LifeTech). Lentiviral particles were generated (ViraPower, Thermofisher Scientific) and used to transduce HEK-293 cells (Sigma Aldrich). Pools of stable transformants were selected with 8 μg/mL Blasticidin. In addition, SH-SY5Y cells (ATCC) were transduced with a CHT construct tagged with GFP at the C-terminal (Origene, PS100071) or a FAP tag at the N-terminus (Sharp Edge Laboratory). Both stable pools and single clones were expanded. HEK-293 cells were cultured in DMEM High glucose (Gibco # 21969-035) supplemented with 10% FBS and 4 mM Glutamine (Thermofisher Scientific). SH-SY5Y cells were cultured in DMEM:F12 with Glutamine (Gibco # 11320-033) supplemented with 15% FBS and 1x NEAA (Thermofisher Scientific).

### Field-based approach to identifying novel CHT modulators

The Cresset field based virtual screening tool, Blaze (formerly called FieldScreen), (Cheeseright et al., 2008, 2009) was utilized to search the full Pfizer compound screening collection to identify compounds similar to literature CHT positive allosteric modulator (PAM) MKC-351/coluracetam (Takashina et al., 2008a,b) or CHT negative allosteric modulator (NAM) ML-352 (Ennis et al., 2015). Similarity was assessed using 50% 3D electrostatic and hydrophobic properties (Cheeseright et al., 2006) and 50% shape (Grant et al., 1996). This field was then used as a template to virtually screen the Pfizer file for additional compounds with a similar field and potentially related biological activity. For each virtual screening run, the top 500 compounds from the Pfizer compound collection, based on Blaze score, were selected. The set of 1,000 compounds identified from the PAM and NAM virtual screening campaigns was further filtered based on compound availability and removal of chemically unattractive groups (Stepan et al., 2011) to generate a test-set set of 887 compounds.

### Chemogenomic compound library

The Pfizer Chemogenomic Library (CGL) contains 2,753 selective small molecules covering 1,043 distinct biological targets (Jones and Bunnage, 2017). The CGL was created to support phenotypic screening with the purpose of expediting target identification. A hit from the set suggests the annotated activities of that pharmacological agent may be involved in perturbing the observable phenotype. Multi-parameter optimization was used in the creation of the library to ensure appropriateness of molecules for cell-based screening (including assessments of permeability, solubility, cytotoxicity and selectivity). CGL compounds were selected on their potency against their primary annotated target at a concentration equal to or less than 500 nM where possible.

### SURFE^2^R

#### Preparation of CHT containing membrane fragments

A single cell clone of HEK293 overexpressing CHT (CHT-WT4) cells was used to generate membrane fragments to assess on the SURFE^2^R platform. Cells were grown to 80% confluence and harvested. The membrane fraction was collected by ultracentrifugation and the plasma membranes were isolated by density gradient centrifugation (Schulz et al., 2008).

#### Thiol-coating of the sensors

All experiments were performed on the SURFE^2^R N1 device and the matching N1 sensor blanks (Nanion Technologies GmbH, Munich). The sensor blanks include a 3 mm diameter gold surface inside a small well coated by incubating 50 μL of 0.5 mM 1-octadecanethiol dissolved in isopropanol for 30 min, rinsed once with isopropanol and twice with water (ddH_2_O) and dried for 30 min at room temperature.

#### Preparation of the solid supported membrane (SSM)

Buffers were prepared according to the following schedules: Potassium buffer–30 mM HEPES, 5 mM MgCl_2_, 140 mM KCl, pH 7.4 with KOH; Sodium buffer—30 mM HEPES, 5 mM MgCl_2_, 140 mM NaCl, pH 7.4 with NaOH; Choline buffer—Sodium buffer + 100 μM Choline Chloride. 7.5 μg/μL DPhPC (Avanti Polar Lipids) was dissolved in n-Decane. 1.5 μL were added onto the thiol-coated gold surface. Immediately, 80 μL of potassium buffer was added carefully. The CHT membrane preparation was diluted 1:10 with potassium buffer and sonicated. Eight microliter were added directly onto the SSM by submerging the pipette into the buffer and the sensors were centrifuged for 30 min at 2,200 × *g*. The quality of the SSM was controlled by determination of conductance σ and capacitance *C*. The SURFE^2^R N1 device includes default functions to perform these measurements. Sensors should have a conductance below 3 nS and capacitance below 20 nF.

#### Electrophysiological measurements

Prepared sensors were inserted into the faraday cage of the SURFE^2^R N1 device and buffers applied via its automatic perfusion system, allowing rapid buffer exchange in a continuous liquid flow. The following buffer addition sequence was used for all experiments: Sodium buffer was applied for 2 s with a flow rate of 200 μl/s to establish a sodium gradient over the membrane. Retaining the flowrate of 200 μl/s, choline buffer was applied for 2 s and washed out again by sodium buffer (2 s). During those 6 s the current response was recorded. At the end of the recording the sensor was rinsed thoroughly with potassium buffer. Only sensors generating current signals with amplitude higher than 100 pA were used for experiments. Signals were used for evaluation after the first activation, which showed greater amplitude. A baseline subtraction was performed using sodium free buffers.

#### Data analysis

Raw data were exported as ASCII files, analysis was performed using the scientific graphing and data analysis program IGOR Pro 6 (WaveMetrics, Portland, USA). The evaluated peak currents were determined using a peak detection algorithm. For every average value, results from different sensors were compared. The errors bars indicate the standard error of the mean. Concentration response relationships for inhibition and apparent affinity were obtained by perfusion of the sensors with increasing compound concentrations. Inhibitors were added to all three buffers. Data were normalized to the maximum amplitude and described by fitting to a Hill function (Boyman et al., 2009; Ottolia et al., 2009).

### Radiometric choline uptake assay

Uptake of [^3^H]Choline (American Radiochemicals; ART 0197; 1,000 Ci/vial; Specific Activity 60–90 Ci/mmol) was measured in HEK cells expressing the high affinity choline transporter (CHT), clone WT4. Cells were plated at 50,000 cells /well in 200 μL culture media on a PDL coated Cytostar-T 96 well plate (Perkin Elmer) 24 h prior to assay. On the day of assay, following two washes and a 30 min pre-incubation at 37°C in 50 μL Na(−) free buffer (0.54 mM KCl, 1.3 mM CaCl_2_, 0.53 mM MgCl_2_, 0.4 mM MgSO_4_, 0.37 mM KH_2_PO_4_, 240 mM Sucrose, 4.4 mM Tris-phosphate, 5.5 mM Glycine, 5.6 mM D-Glucose; pH 6.5 with KOH), 25 μL 4X control or test compounds in Na(−) buffer were added to the cells. Hundred and zero percent controls were defined with 10 μM Staurosporine and DMSO respectively. Final DMSO concentration was 0.25%. Following a 30 min incubation at 37°C, 25 μL [^3^H]Choline diluted to 320 nM (80 nM final concentration) was added in Na(+) buffer (0.54 mM KCl, 1.3 mM CaCl_2_, 0.53 mM MgCl_2_, 0.4 mM MgSO_4_, 0.37 mM KH_2_PO_4_, 138 mM NaCl, 0.28 mM Na_2_HPO_4_, 5.5 mM Glycine, 5.6 mM D-Glucose; pH 6.5 with KOH). Plates were sealed with TopSeals (Perkin Elmer) and incubated in darkness for 3 h at room temperature before reading using a Wallac Microbeta, 1 min/well. The assay window was typically 3-fold signal to background ratio. A number of datasets fell outside of assay acceptance criteria (defined as signal to background ratio >3, individual assay plate Z prime (also known as z-factor) value >0.3 and EC_50_ of a standard compound within the expected range) and were therefore not included in the analysis.

### Mass spectrometric choline uptake assay

All LC/MS analyses were performed on a Sciex 6,500 triple quadrupole tandem mass spectrometer in positive electrospray ionization (ESI) mode. Other instrumentation consisted of Shimadzu LC-20AD pumps and an Apricot Design Dual Arm Autosampler (ADDA). Liquid chromatography was performed on a Waters Atlantis HILIC column (10 × 2.1 mm, 3μ). Mobile phase A consisted of water with 0.1% formic acid and mobile phase B consisted of water containing 0.1% formic acid and acetronitrile containing 0.1% formic acid (50:50). The flow rate was 0.6 mL/min and the gradient was as follows; hold at 100% B for 5 s and switch to 100% A for 10 s and return to 100% B for 5 s. Data were acquired with Analyst version 1.6. Quantitative analysis was performed in the multiple reaction monitoring (MRM) mode using MultiQuant software version 2.1. The MRM transitions monitored were m/z 113/60 m/z for choline-D9 (deuterated choline chloride-(trimethyl-d9), Sigma Aldrich) and 108/60 m/z for the internal standard, choline-D4 (deuterated choline chloride-1,1,2,2-D4, Sigma Aldrich).

### Antibody staining

A single cell clone of HEK293 overexpressing CHT (CHT-WT4) cells was grown in 96 or 384 well format to 50% confluency. Media was removed and cells were washed once with HBSS (+/+). 1 μg/mL Anti-FLAG antibody (F3165, Sigma) was diluted in HBSS (+/+) and added to the cellular monolayer for 30 mins. After incubation, cells were washed three times with HBSS (+/+) followed by incubation with 1:5,000 AF488 labeled secondary antibody (LifeTech) diluted in HBSS (+/+) for 30 min. After three washes, cells were fixed with 4% paraformaldehyde in HBSS (+/+) for 15 min. Nuclei were counterstained with the nuclear dye Hoechst. All images were captured and analyzed on an epifluorescent microsope or Cellomics platform.

## Results

### Stable cell line generation and characterization

Historically, animal-derived materials such as synaptosomes and primary culture have been used to study CHT function. We sought to move away from these approaches in accordance with UK NC3R guidelines. We generated and characterized a range of stable recombinant cell lines in both HEK-293 and SH-SY5Y backgrounds as neuronal-like cell lines in order to assess CHT transporter function for drug discovery. In the HEK-293 background, stable pools with either wild-type (WT) or mutant (LV-AA) CHT constructs were expanded and used to generate genetically homogenous clonal lines using single cell flow cytometry. Pools were used for initial radiometric assay development which facilitated subsequent selection of the CHT-WT4 line over other clones. This was done on the basis of greater assay window for inhibition and activation of [^3^H Choline] uptake (using 1 μM HC-3 and 10 μM STS respectively; Supplementary Figure 1) and data reproducibility. Interrogation of CHT-WT4 revealed overexpression of the codon-optimized CHT transcript but not endogenous *SLC5A7* transcript as measured by qPCR but no differential expression of other choline transporters (*SLC6A12*/BGT1, *SLC44A1-4/*CLT1-4) (Supplementary Figure 2A). Similarly, there is no differential expression of other molecules required for acetylcholine synthesis (*CHAT*), transport (*SLC18A3*/vAChT) or hydrolysis (*ACHE*); nor in muscarinic and nicotinic acetylcholine receptor subunits (Supplementary Figures 2B,C). Parallel experiments were performed on the SH-SY5Y stable cell lines which overexpressed the codon-optimized CHT-GFP fusion transcript in addition to some basal *SLC5A7* expression in parental cells in addition to *ACHE, SLC18A3*, and *CHAT* expression (Supplementary Figures 2D–F). These cell lines were the basis for assessing a range of assays to identify and characterize molecules that increased CHT-mediated transport (Table 1).

**Table 1:** **Method comparison**.

**Type of readout**	**Assay**	**Pros**	**Cons**	**Notes**
Direct measure of transport function	[Choline] uptake into synaptosomes	Gold standard, decades of literature precedence	Radioactive Low throughput Uses *ex-vivo* preparations, does not uphold UK NC3R ideals	Data not shown
	[Choline] uptake detected by scintillation proximity assay (SPA)	Recapitulates gold-standard inhibitor data Potential for further assay development (e.g., 384 well, automation)	Radioactive Less sensitive than gold standard assay, compressed assay window Low-to-medium throughput Requires adherent cells	Data shown for HEK293 CHT-WT4; comparable data sets not shown for HEK293 CHT-LVAA and SH-SY5Y CHT-GFP cell lines
	D9-choline uptake detected by LC/MS (liquid chromatography/mass spectrometry)	Recapitulates gold-standard inhibitor data Reasonable throughput 384 well format possible Increased sensitivity compared to radiometric format Saturable—can measure kinetics, mechanism etc	High throughput options for large compound collections may be limiting	Data generated for HEK293 CHT-WT4 only Potential to be modified for metabolic fate studies (see below) and for *in-vivo/ex-vivo* approaches (e.g., MALDI-Ach; Shariatgorji et al., 2014)
	Brominated choline detected by X-ray fluorescence	Potentially comparable to D9-choline LC/MS approach	Suitable ligand needs to be identified	Did not fully assess format
Electrogenic measurement of transport function	Nanion SURFE^2^R to detect membrane potential changes	Recapitulates gold-standard data Analogous to validated approach (Ennis et al., 2015)	Low-to-medium throughput Requires large amounts of membrane preparation	Data shown for HEK293 CHT-WT4
Transporter localization	FLAG-tagged live cell labeling	Potential for mechanistic transporter assessment	Low throughput Less sensitive than gold standard assay Challenging to generate IC/EC_50_	See discussion
	FAP (fluorogen activated peptide) tagged assessment	Potential for detailed transporter mechanism assessment	Requires generation of custom cell line (performed under contract by Sharp Edge Laboratories)	Preliminary data suggests custom cell line does not transport [choline] despite apparent compound effects on transporter localization (data not shown)
	Hemicholium-3 binding assay	Literature precedence Potential to generate B_*max*_data	Requires large amounts of radioactivity Potentially confounding observations given that inhibitor (HC-3 and ML-352) treatment increases cell surface localization	See discussion
Other *in vitro* assays	Metabolic fate of transported D9-labeled choline	Non-radioactive	Low throughput	Potential for proof-of-concept experiments to test key hypothesis that increasing CHT function impacts on ACh resynthesis and release Preliminary experiments suggest feasibility of approach (data not shown)
	*In vitro* acetylcholine quantitation assay	Non-radioactive	Low throughput Highly variable and not very sensitive	Gold-standard uses radiolabeled choline which gets taken up by presynaptic terminals and presumably used to synthesize acetylcholine Preliminary data suggests room for improvement
	Chemical biology approaches	Literature describing tagged choline mimetics informing design of tools (fluorescent, biotinylated and clickable tools)	Unclear how much tolerance transporter has for chemical substitution or other substrates Low throughput	Preliminary experiments failed to recapitulate literature approaches
	Slotboom transport dynamics assay	Detailed assessment to inform on structure and transport rate (Erkens et al., 2013)	Low throughput and labor intensive Ideally requires crystal structure information Non-radioactive	Did not assess
*In-vivo* impact of tool compounds (ideally at least 2 chemotypes) on choline clearance, ACh resynthesis and release	Ileum preparation	Could inform on probability of parasympathetic side effect Proven utility for assessing inhibitors	Novel approach, requires further method development	Preliminary data using overexpressing mouse model did not see any effect with genotype or compounds—but we did not observe increased choline uptake in synaptosomes from overexpressing mice (data not shown)
	Amperometry in brains of anaesthetized animals	Previous literature suggests feasibility of approach (Parikh and Sarter, 2006)	Labor intensive, would require prior compound triaging	Preliminary experiments suggests recapitulation of literature data (not shown)
	Acute slice culture	Potential for more throughput (parallel assessment in slices)	Assay development required	
	Behavioral assessment in relevant animal model	Potential for disease relevance and/or phenotype relevance	Labor intensive and low throughput	dSAT (sustained attention task in presence of distractor) task likely to be most informative (Parikh et al., 2013); perform vs. AChEI
*In vivo* safety assessment	Parasympathetic side effect assessments	Methods exist for assessment e.g., cardiovascular telemetry, urination, defecation (metabolic cages), gastrointestinal motility, functional observational battery		Perform vs. AChEI

### Assessment of transporter function using membrane potential has utility as an orthogonal assay format

**Figure 1:**
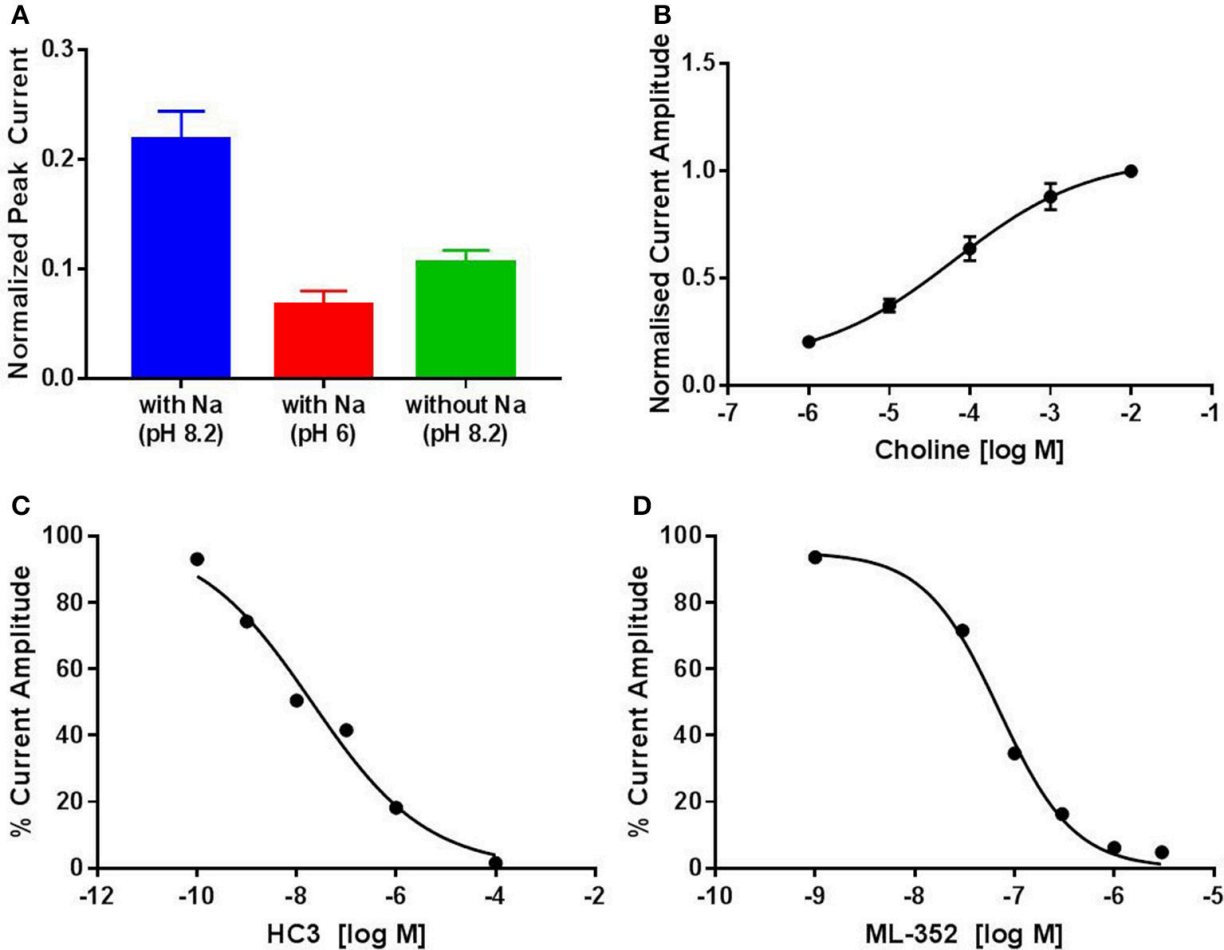
**Solid supported membrane based assays to evaluate CHT transport function. (A)** Na^+^ and pH dependent effects on CHT. Normalized peak current is increased with higher pH. Compare pH 8.2, blue, vs. pH 6.0, red (*p* = 0.0002, 1-way ANOVA). Absence of Na^+^ leads to a decrease in current. Compare presence of Na^+^, blue, with absence of Na^+^, green (*p* = 0.0015, 1-way ANOVA). (*N* = 11). Error bars represent ± standard deviation. **(B)** Apparent choline affinity is measured by application of solution containing differing choline concentrations as indicated. EC_50_[choline] 25 ± 6 μM (*N* = 10). Hill coefficient: 0.5. **(C**,**D)** Normalized peak current was decreased by application of different concentrations of **(C)** HC-3: IC_50_ 20.4 nM (*N* = 12) and **(D)** ML-352: IC_50_ 70.1 nM (*N* = 13).

CHT is sodium (Na^+^) and chloride (Cl^−^) dependent and requires a negative membrane potential; hence, alterations in membrane potential can be used as a measure of CHT-mediated transport. Indeed, Blakely and colleagues reported the discovery of ML-352, a novel non-competitive inhibitor and STS as a positive modulator of CHT using a membrane depolarized assay (Ruggiero et al., 2012; Ennis et al., 2015). Building on these observations, we evaluated the SURFE^2^R™ platform (Nanion Technologies), as a solid supported membrane (SSM) based electrophysiology platform to assess electrogenic transporter activity (Bazzone et al., 2013; Barthmes et al., 2016). Membrane fragments containing the protein of interest are immobilized on a gold electrode several millimeters in diameter to facilitate detection of transporter currents. Synchronous activation of the transport proteins is triggered by rapid application of a substrate-containing buffer. During electrogenic transporter action, charge accumulates in the membrane fragments which directly correlate with the measurable current on the gold electrode and thus enables flexible, robust real time measurement of transporter activity. Membrane preparations are essentially “cell free” preparations minimizing trafficking impacts such that any effect seen is likely to be a result of direct action of compound or other treatment on the transporter. However, it should be noted that the comparatively low proportion of CHT proteins at the plasma membrane may be a limiting factor for assays made using the SURFE^2^R™. In the first instance, we demonstrated that the Na^+^ and pH dependence of CHT mediated choline transport could be recapitulated using membrane preparations from CHT-WT4 (Figure 1A). Furthermore, we were able to saturate the assay with an apparent *K*_*a*_ of 25 μM (Figure 1B) and noted the measured signal was stable over time (Supplementary Figure 3). We observed inhibition of current with HC-3 (Figure 1C) and ML-352 (Figure 1D) with IC_50_ estimates of 20.4 and 70.1 nM respectively (Table 2). However, we were not able to detect any effect of staurosporine (STS) or MKC-231.

**Table 2:** **Tool compound data**.

**Compound**	**Structure**	**CHT Pharmacology**	**Origin**	**3H-choline uptake**			**D9-choline uptake**			**SURFE**^**2**^**R™**		
				**% effect 10 μM**	**% effect 1 μM**	**IC_50_ or EC_50_(M)**	**% effect 10 μM**	**% effect 1 μM**	**IC_50_ or EC_50_(M)**	**% effect 10 μM**	**% effect 1 μM**	**IC_50_ or EC_50_(M)**
HC-3	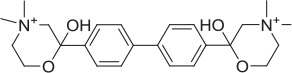	Inhibitor	Apparsundaram et al., 2005			1.16 E-07			5.6 E-09			2.04 E-08
ML-352	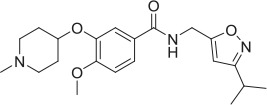	NAM	Ennis et al., 2015			5.49 E-07	104.1	96.3	4.19 E-08			7.01 E-08
STS	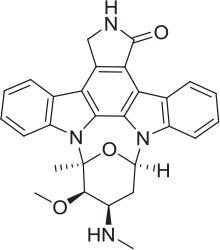	PAM	Ruggiero et al., 2012			1.7 E-06	87.9	48.4	5.07 E-07			n.d
MKC-231	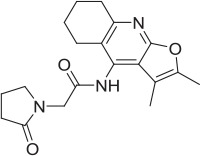	PAM	Takashina et al., 2008a,b			n.d.			n.d.			n.d.
1	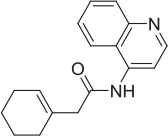	PAM	MKC-231 seed, field-based virtual screen			2.75 E-07						
2	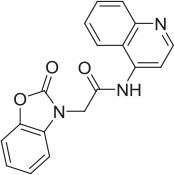	PAM	MKC-231 seed, field-based virtual screen			4.16 E-07						
3	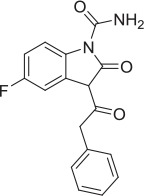	PAM	MKC-231 seed, field-based virtual screen			4.51 E-06						
4	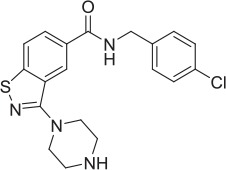	NAM	ML-352 seed, field-based virtual screen			1.54 E-06	118.1	12.3	3.13 E-06			
5	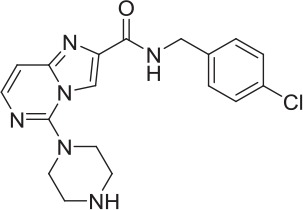	NAM	ML-352 seed, field-based virtual screen			6.70 E-07						
6	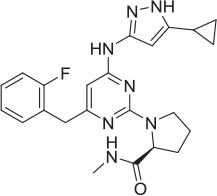	PAM	Chemogenomics library	23.6	3.6		91.6	60.8	2.05 E-06			
7	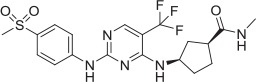	PAM	Chemogenomics library	26.7	−2.4		80.8	46.4	2.49 E-06			
8	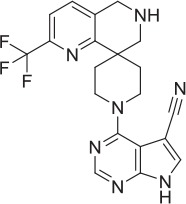	PAM	Chemogenomics library	52.6	9.0		80.2	24.5	5.10 E-06			
9	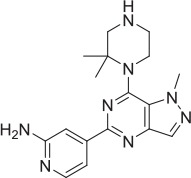	PAM	Chemogenomics library	100.0	24.4		64.0	30.5	5.76 E-06			

*n.d. not detected*.

### Radiometric assessment of CHT mediated transport function was not suitable for screening

**Figure 2:**
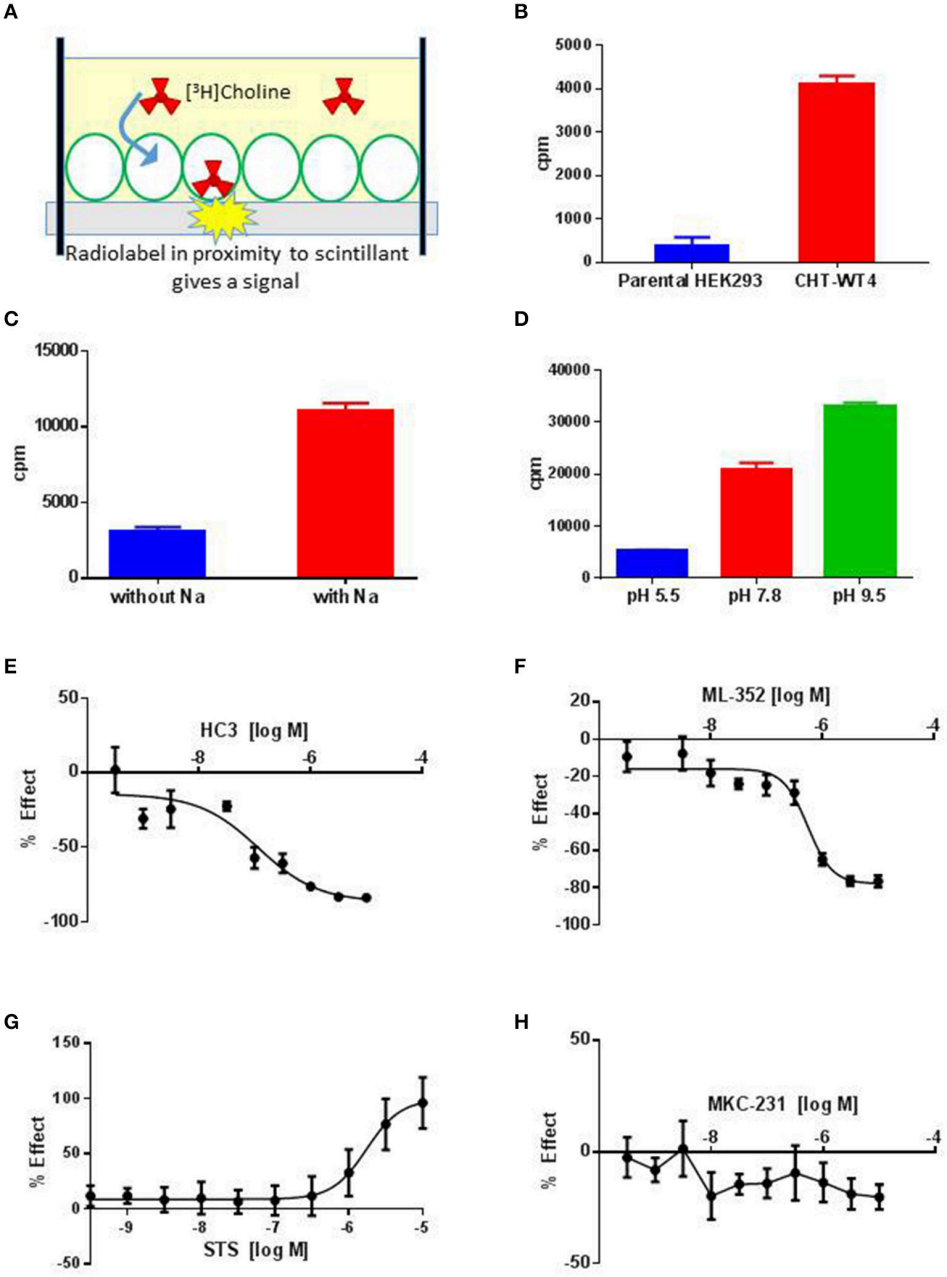
**Radiometric assay to evaluate CHT transport function. (A)** Schematic showing principle of the radiometric assay format. A monolayer of CHT expressing cells (green outline) are grown on plates with scintillant embedded in the base. Uptake of [^3^H] Choline (red) by cells brings the radioligand in proximity to the scintillant giving rise to a signal that can be quantified. **(B)** Specific [^3^H] Choline uptake (plotted as cpm, counts per minute) observed in recombinant HEK293 cell line overexpressing wild-type CHT (Clone 4, CHT-WT4, red) compared to the parental HEK293 background (blue) (*N* = 8–24; *p* < 0.0001, unpaired *t*-test). Bars represent ± SD. **(C)** [^3^H] Choline uptake (plotted as cpm, counts per minute) is measured in the presence (red) or absence (blue) of sodium (Na) in the CHT-WT4 cell line. A clear increase in choline uptake is observed in the presence of sodium (*p* < 0.0001, unpaired *t*-test, *N* = 18). Bars represent ± SD. **(D)** [^3^H] Choline uptake (plotted as cpm, counts per minute) is measured at pH 5.5 (blue), 7.8 (red) and 9.5 (green). Uptake increases with increase in pH (Multiple comparisons performed with 1-way ANOVA. pH 5.5 vs. pH 7.8 *p* = 0.0009; pH 5.5 vs. pH 9.5 *p* < 0.0001; pH 7.8 vs. pH 9.5 *p* = 0.019. *N* = 2). Bars represent ± SD. **(E–H)** 10-point dose response curves to generate IC_50_ or EC_50_ estimates performed for HC3, IC_50_ 116 nM **(E)**, ML-352, IC_50_ 549 nM **(F)**, STS, EC_50_ 1.7 μM **(G)**, MKC231 (no apparent effect) **(H)**; (*N* = 8). Bars represent ± SD for each data point. Cpm, Counts per minute; refers to uptake of [^3^H] Choline.

SSM based approaches have utility as an orthogonal approach but may be currently limited by throughput; additionally we were also unable to observe positive effects of STS on CHT-related currents (see Section Discussion). A preponderance of literature precedent for measuring high affinity choline uptake (HACU) utilizes a radiometric assay to measure [^3^H]Choline transport in preparations. In order to measure CHT-mediated transport activity, we established a radiometric assay based on a measure of proximity-induced scintillation in recombinant cell lines (Figure 2A) which had increased throughput compared to traditional approaches. We observed an increase in [^3^H]Choline uptake in CHT-WT4 cells compared to the parental HEK293 background (Figure 2B) together with a concentration-dependent increase in [^3^H]Choline uptake (Supplementary Figure 4A). In addition, we recapitulated literature observations of sodium dependency (Figure 2C), consistent with known Na+ dependent choline co-transport activity as well as recapitulating pH dependency observations (Figure 2D) (Okuda et al., 2000). A number of parameters were assessed in assay development including cell seeding density, time of assay post-seeding, plate coating, buffer composition, read time and choline concentration. We noted that choline starvation and use of a sodium gradient and manipulating pH (decreasing pH increases STS response) improved assay window. We also observed the STS mediated enhancement of [^3^H]Choline was stable over time and there was reduced variability which led to the selection of a 3 h time point for screening purposes (Supplementary Figure 4B). However, a complete saturation curve could not be generated as the 3H-[choline] contained ethanol as a diluent which was toxic to the cells at higher concentrations; a limitation of this assay format. We proceeded to assess reported tools in this assay which was capable of reading out on both inhibitory and stimulatory activities (Table 2). Inhibition of CHT mediated transport was seen with the classic inhibitor hemicholinium-3 (HC-3) with an IC_50_ estimate of 116 nM (Figure 2E) in addition to the recently described negative allosteric modulator ML-352 with an IC_50_ estimate of 549 nM (Figure 2F) which is in broad agreement with literature observations (Okuda et al., 2000; Ennis et al., 2015). We further recapitulated the reported stimulatory effect of staurosporine (STS) on CHT-mediated transport (Ruggiero et al., 2012) with an EC_50_ estimate of 1.7 μM in CHT-WT4 cells (Figure 2G) but no apparent effect in CHT-LVAA pools (data not shown). We were unable to satisfactorily demonstrate potentiation of CHT transport with STS in SH-SY5Y cell backgrounds due to toxicity. We were also unable to see any impact of positive allosteric modulator MKC-231 (Takashina et al., 2008b) on CHT mediated transport (Figure 2H).

We selected a set of 887 Pfizer compounds selected *via* the Cresset field-based virtual screening technology, (Cheeseright et al., 2007) using seed molecules CHT PAM MKC-351/coluracetam (Takashina et al., 2008b) and CHT NAM ML-352 (Ennis et al., 2015) (Supplementary Figure 4). This library was initially screened in the CHT radiometric assay in a 96 well format at 1 and 10 μM compound concentrations. Actives identified in the 1 and 10 μM single point screens were then assessed in a 10-point dose response format (Table 2). Compounds **1**, **2**, and **3** were confirmed as positive allosteric CHT modulators with EC_50_ estimates of 0.3, 0.4, and 4.5 μM respectively. Compounds **4** and **5**, with EC_50_ estimates of 1.5 and 0.7 μM respectively, were characterized as negative allosteric modulators of CHT function. The Chemogenomic Library (CGL) was also screened in the CHT radiometric assay in a 96 well format at 10 and 1 μM concentrations. From this screening campaign, a number of positive CHT modulator hits (compounds **6-9**, Table 2) were identified. The CHT radiometric assay identified CHT modulators from both the field-based virtual screening campaign and the Chemogenomic Library (CGL) screen. However, as a consequence of the small assay window, inter-assay variation was high and not all datasets fell within the assay acceptance criteria as defined in the Materials and Methods section. This, coupled with our inability to satisfactorily perform saturation analyses, prompted us to assess alternative screening platforms.

### Mass spectrometry assessment of transport function enabled screening to identify novel positive modulators

**Figure 3:**
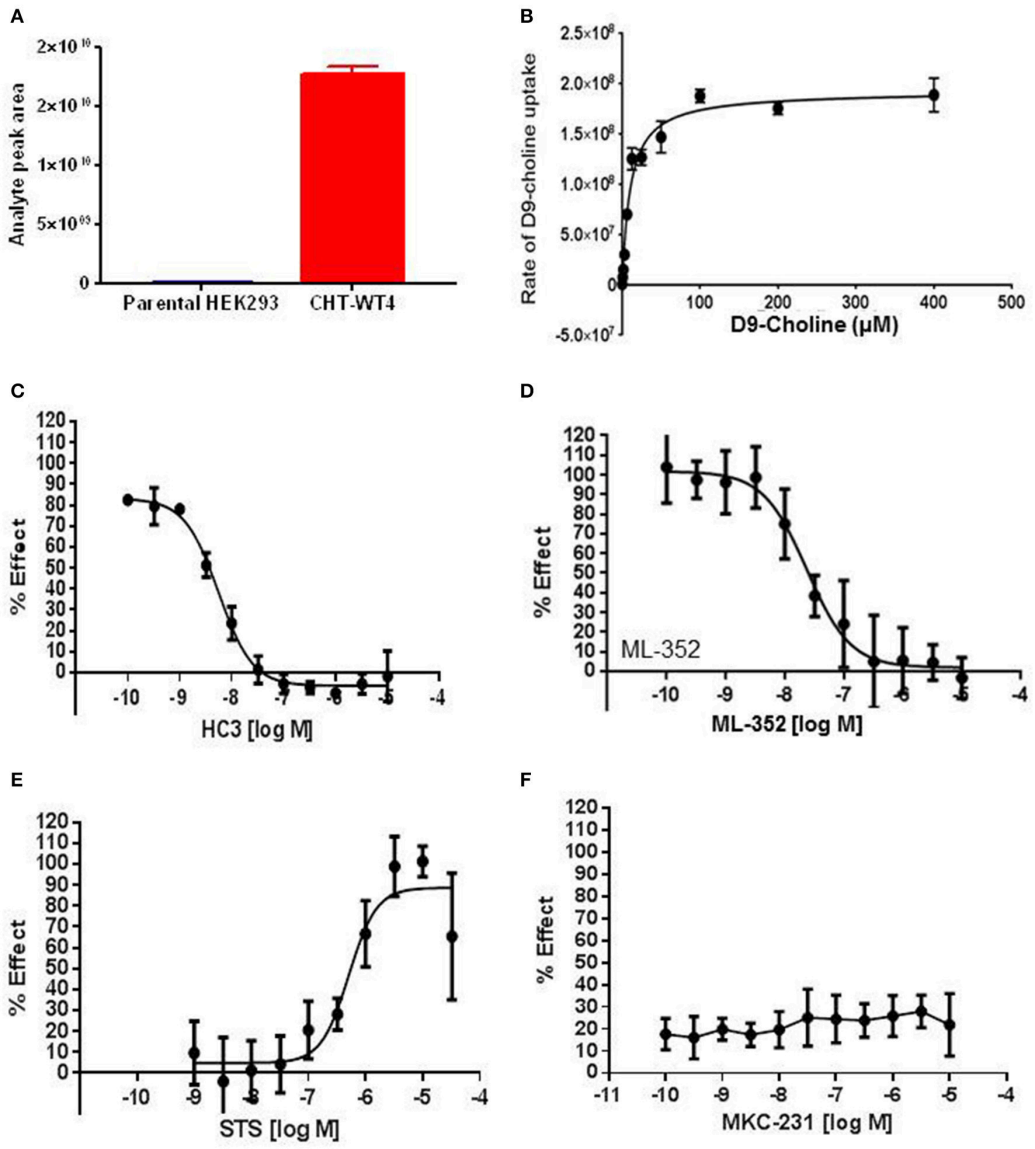
**Mass spectrometric assay to evaluate CHT transport function. (A)** Specific D9-Choline uptake observed in recombinant HEK293 cell line overexpressing wild-type CHT (Clone 4, CHT-WT4, red) compared to the parental HEK293 background (blue) (*p* < 0.0001, unpaired *t*-test. *N* = 3). Bars represent ± SD. **(B)** Saturation of D9-Choline uptake in the presence of increasing concentration of D9-Choline (*N* = 2). Bars represent ±SD. **(C–F)** 10-point dose response curves to generate IC_50_ or EC_50_ estimates performed for HC3, IC_50_ 5.6 nM **(C)**, ML-352, IC_50_ 41.9 nM **(D)**, STS, EC_50_ 507 nM **(E)**, MKC231, no apparent effect **(F)** (*N* = 3). Bars represent ±SD for each data point.

As highlighted in Section Radiometric assessment of CHT mediated transport function was not suitable for screening, given the challenges of our radiometric assay for high throughput screening, alternative methods for measuring choline uptake suitable for screening compound libraries were sought. We elected to focus on measuring transport of a stably labeled form of choline (deuterated choline chloride-(trimethyl-d9); D9-choline) coupled with mass spectrometric based quantification (Koc et al., 2002; Shariatgorji et al., 2014; Iwamoto et al., 2016). Using this method, we observed a clear increase in D9-choline uptake in HEK293 CHT-WT4 recombinant cells compared to the parental background (Figure 3A). Critically, we were also able to saturate transporter function using this method which led us to determine a *K*_*m*_ of 7.0 μM (Figure 3B), consistent with literature reports (Okuda and Haga, 2000; Okuda et al., 2000). Further validation of the mass spec assay using our tool compounds revealed the capacity of the assay to report on both inhibitory and potentiation activities. Pleasingly, we observed a good agreement with obtained IC_50_ estimates for HC-3 (5.6 nM) and ML-352 (41.9 nM); and EC_50_ estimates of STS (507 nM) with those obtained in the radiometric assay and in the published literature (Figures 3C–E and Table 2). Again, we saw no apparent effect of MKC-231 (Figure 3F), consistent with lack of activity in all other assay formats. We successfully miniaturized the assay to a 384 well format with consistent z prime >0.5 (Supplementary Figure 6). As a proof of concept study, we screened the Chemogenomic Library (CGL) at 1 and 10 μM with DMSO as the negative control and 10 μM STS as the positive control. A number of hits with either inhibitory or stimulatory activities were identified, and these hits confirmed with 10 point EC_50_ dose response follow-up. As highlighted in Table 2, compounds **6**-**9**, flagged as CHT PAMs in the single point radiometric assays, were identified as positive modulators in the mass spectrometry assay. These four compounds, all kinase inhibitors, were selected for further assessment in orthogonal assay formats (Tables 1, 2, Section Live cell staining reveals effect on transporter localization with compound treatment).

### Live cell staining reveals effect on transporter localization with compound treatment

**Figure 4:**
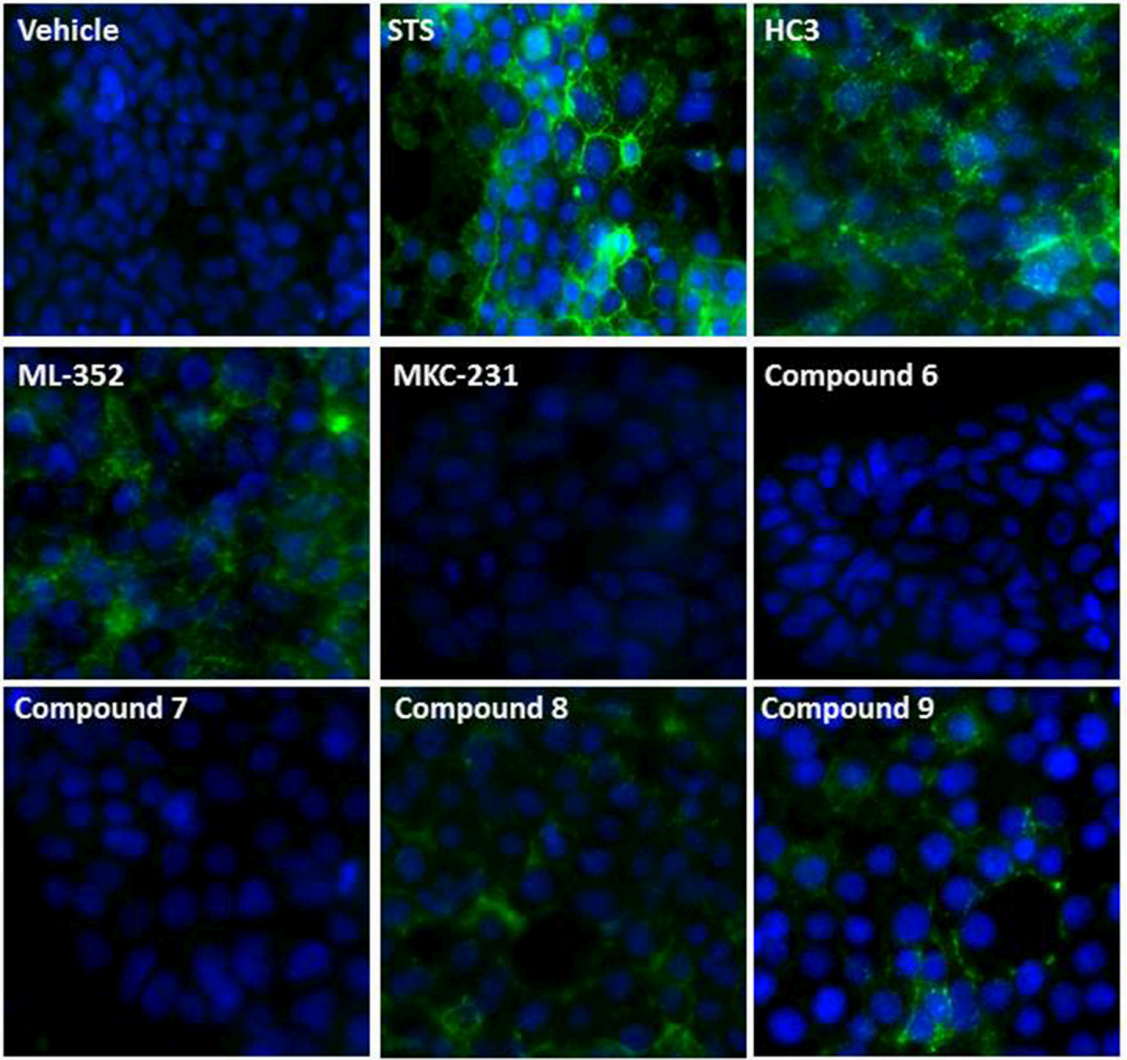
**Effect of compounds on CHT localization**. Representative images showing effect of compounds on cell surface expression of CHT measured by immunocytochemistry of the N-terminal FLAG tag (green) in live CHT-WT4 cells. Nuclei are counterstained with DAPI (blue). Vehicle represents 0.1% DMSO and indicated compounds were used at a concentration of 10 μM. Images were captured at 40x magnification.

CHT function has been proposed to be tightly regulated through localization with a minority of transporters present on the presynaptic membrane (<10% of the total population; Ferguson and Blakely, 2004; Ribeiro et al., 2006, 2007; Black and Rylett, 2012). Thus, understanding compound effects on CHT density and activation state in the synaptic membrane may aid interpretation of the effects on cholinergic neurotransmission. Conventional methods such as cell surface biotinylation present challenges in terms of the requirement of large quantities of cells and low throughput. The predicted topology of CHT indicates 13 transmembrane domains, with an extracellular N-terminus and intracellular C-terminal tail (Okuda et al., 2012). Our CHT overexpression construct contains a FLAG tag on the N-terminus which we sought to utilize with antibody detection in non-permeabilized cells. However, we found that fixation of cells with PFA or methanol gave rise to some permeabilization and labeling of the internal CHT pool as indicated by immunostaining for the V5 epitope tag at the intracellular C-terminus (Supplementary Figure 7A). We therefore moved to live cell immunolabeling to reduce artifacts. We used the CHT-LVAA pool vs. CHT-WT4 cells to assess the effectiveness of this approach in the first instance as this mutant protein has been reported to exhibit increased cell surface localization through a decrease in endocytosis (Ferguson et al., 2003; Ribeiro et al., 2005, 2007; Ruggiero et al., 2012; Supplementary Figure 7B). We proceeded to investigate the effect of compound treatment in more detail. Cells were incubated with compounds at 1 or 10 μM for 1 h before immunolabeling and visualization. Interestingly, we observed an increase in cell surface localization with HC-3, ML-352 and STS treatments whereas again, MKC-231 had no apparent effect (Figure 4). We further tested compounds 6-9, initially identified by screening in the radiometric and mass spectrometry assays at 1 and 10 μM, and noted that compounds 8 and 9 both increased cell surface localization of CHT but compounds 6 and 7 appeared to have no effects based on qualitative observations (Figure 4).

## Discussion

In this manuscript, we report on two overlapping areas of research: (1) seeking to develop novel approaches for identifying and characterizing molecules that positively modulate high affinity choline transport (ligand transport modulator), and (2) the discovery of novel molecules that could be used as tools or starting points for further medicinal chemistry efforts. It is worth noting here that the methods we describe in this manuscript have broader potential applications with respect to the field of transporter biology.

Classical approaches to measuring HACU as a primary function of CHT involve measuring the transport of radioactively labeled choline in a relevant substrate. We were unable to find a good immortalized source of human cholinergic neurons suitable for large scale culture though there are some putatively suitable murine lines (e.g., HT22) or stem cell derived cholinergic models (Liu et al., 2013; Ho et al., 2016). We generated stable, clonal lines in HEK-293 (a robust and well-understood line with properties reminiscent of immature neurons) and SH-SY5Y (neuroblastoma line which has been reported to differentiate toward a cholinergic phenotype with retinoic acid) backgrounds. These lines were characterized with respect to cholinergic expression and function; we believe they serve as useful tools for primary screening and other throughput approaches but given the known caveats with overexpressing cell lines, we would advocate further corroboration in a more “native” context.

Radiometric choline uptake, a direct assessment of the transport function and detected by scintillation proximity assay is a method we have previously used successfully [52, 53]. Assay development enabled us to refine parameters to increase assay window including the use of sodium free buffer prior to substrate and compound exposure to enhance the gradient and pH manipulation. Two sets of compounds were screened in the radiometric choline uptake assay. The first set was selected using the field-based approach from Cresset (Section Field Based Approach to Identifying Novel CHT Modulators, Supplementary Figure 5). If, as is the case for the choline receptor, structural information describing key ligand/protein interactions between a bound active ligand and a protein of interest is not available to act as a start point for virtual screening, an alternative approach is to use a ligand-based method. The Cresset approach computationally describes an active ligand as a 3D electrostatic and shape-based “field” to give a “protein's eye view” of how the compound interacts with the target. This “field” is then utilized as a template to virtually screen compounds collections in order to identify additional compounds with a similar field and therefore biological activity (Cheeseright et al., 2007). As discussed in Sections Chemogenomic Compound library and Radiometric assessment of CHT mediated transport function was not suitable for screening, 500 compounds based on the CHT PAM modulator MKC-351/coluracetam (Takashina et al., 2008b) and 500 compounds based on the CHT NAM modulator ML-352 (Ennis et al., 2015) were selected for screening from the Pfizer screening collection, based on their field scores. We identified a number of positive and negative CHT modulators in our experiments (Table 2). Positive allosteric modulators **1**-**3** were identified from the CHT PAM modulator MKC-351 and negative allosteric modulators **4**-**5** were identified from the CHT NAM modulator ML-352. Compounds **1**-**5** illustrate the value of the field-based approach, with a more varied range of active chemotypes identified than those that would be generated from a simple substructure or 2D similarity search. It is important to note that the original observations for the CHT PAM modulator MKC-231 were made in the AF64A-treated rat hippocampal synaptosomes with no impact of MKC-231 treatment on HACU or HC-3 binding with vehicle treatment (Takashina et al., 2008a,b). Consistently, we did not observe any impact of MKC-231 treatment in any of our approaches which were performed in the absence of AF64A treatment.

The Pfizer Chemogenomic Library (CGL) comprises approximately 2,753 historical compounds covering 1,043 distinct mechanisms (thus testing each mechanism multiple times) and was initially designed to support phenotypic screening assays to help delineate which mechanisms may be playing a role in the observed end point. Pleasingly, we identified compounds with either inhibitory or stimulatory activities. The initial stimulatory compound hits were further confirmed in 10 point dose response curves. Compounds **6-9** are examples of kinase inhibitor actives from the Pfizer Chemogenomic Library (CGL) screen highlighted in Table 2. Literature reports show that CHT activity at the plasma membrane and subcellular trafficking/internalization via endosomal compartments is tightly controlled at least in part by post-translation mechanisms (PTMs) with canonical consensus sequence motifs in the primary amino acid sequence for phosphorylation and ubiquitylation; motifs also exist for dimerization and clathrin-mediated endocytosis (reviewed in Ferguson and Blakely, 2004; Black and Rylett, 2012). Our observations raised the intriguing possibility that these compounds may be exerting effects through modifying CHT PTMs and its localization accordingly. Compounds **6**-**9** were selected for further characterization using the live cell antibody labeling to assess their impact on CHT cell surface localization. Of the four compounds, two were found to have no apparent effect on CHT localization on the cell surface (compounds **6** and **7**), whereas compounds **8** and **9** each elicited a distinct increase in apparent cell surface localization. Interestingly, STS treatment also increased cell surface localization. Additional analysis is required to determine the specific kinase targets/pathways that may be responsible for modulating the phosphorylation state of the CHT transporter and increasing its retention at the cell surface.

In addition to STS treatment, our observations suggest that treatment with inhibitors HC-3 or ML-352 also increase transporter density on the cell surface which in turn invites speculation about conformational changes that block CHT internalization. We therefore suggest caution with respect to interpretation of data from HC-3 binding assays as an indication of active CHT present on the cell surface. Better understanding of CHT conformation dynamics and structural changes corresponding to activity would be invaluable using approaches such as single-molecule FRET (Erkens et al., 2013). Interestingly, preliminary data (not shown) generated under contract by Sharp Edge Laboratories with N-terminal modulation of a CHT construct with a fluorogen activated peptide tag (Snyder et al., 2015; Naganbabu et al., 2016; Plamont et al., 2016) appears to have no effect on trafficking of CHT but potentially interferes with [choline] transport.

Blakely and colleagues report the use of membrane potential changes as a basis for identifying novel compounds to modulate CHT given its electrogenic properties (Ruggiero et al., 2012; Ennis et al., 2015). Data generated with tool compounds in collaboration with Nanion Technologies on the SURFE^2^R™ platform using membrane preparations enabled us to generate direct, sensitive and functional measurements that correlated well with those reported in literature (Table 2). This approach is currently limited in terms of throughput but could be advantageous for orthogonal validation or for deeper study into mechanisms of action. An additional challenge in studying CHT by this method is the relatively low proportion of active transporter on the plasma membrane which may have been addressed to some extent in the literature by using a preparation from an LV-AA mutant form with higher transporter density (Ruggiero et al., 2012; Ennis et al., 2015). It would additionally be interesting to evaluate whether membrane fractions (e.g., plasma membrane vs. vesicles or endocytic machinery) would impact on assay window. Lack of activity of STS on the SURFE^2^R™ platform (Table 2) is further consistent with the hypothesis that STS and STS-like compounds exert their effect through an indirect kinase mechanism that in turn modulates the phosphorylation state of the transporter and increases its retention at the cell surface, as are published data suggesting STS has modulatory effect in striatal but not hippocampal synaptosomes (Ruggiero et al., 2012). Indeed, further biological and pharmacological interrogations around kinase inhibition and phosphorylation motifs and subsequent impact on subcellular localization and trafficking kinetics would be an interesting avenue to explore. Additional studies into the underlying mechanisms of these novel compounds described here include more detailed pharmacology experiments including *K*_*m*_ and *V*_*max*_ determination and relative efficacy of distinct mechanisms with respect to the fate of the transported choline in addition to impact on synaptic vesicle based activities. It is not currently clear whether there is sufficient transporter present at the cell membrane to provide efficacy through direct positive modulation.

The challenges (lack of saturation, requirement for high [^3^H]Choline concentrations and small assay window) associated with screening in the radiometric assay led us to seek alternative approaches. Mass spectrometry has previously been reported as a rapid, sensitive and directly quantitative approach with respect to AChEI (Shariatgorji et al., 2014). In our hands, mass spectrometry appeared advantageous with respect to bidirectional readout, high sensitivity, reproducibility, quantitative output, and compatible with throughput optimisation (e.g., miniaturization, automation). Furthermore, it appears amenable to more detailed kinetic, mechanistic and quantitative studies. We are intrigued by the additional potential to elucidate the fate of transported D9-choline through multiple metabolic routes including detection of released D9-ACh into the extracellular milieu; and associated possibilities for biomarker development. We are similarly captivated by the potential of X-ray fluorescence (Olabisi et al., 2016) with high throughput label-free direct activity-based measurements in normal culture conditions to identify compound effects on CHT overexpressing cells. However, we do not know how much tolerance CHT has for chemical modulation of specific groups and impact on substrate function, for example in order to label a choline mimetic (i.e., bromination of the *N*-methyl group) that can be detected by X-ray fluorescence.

Taken together, we outline a set of approaches suitable for screening to identify and characterize novel small molecules modulating transport function of CHT and selected other solute carriers. Furthermore, we report the discovery of potential positive ligand transport modulators for CHT which warrant further investigation and validation as possible tools and/or seeds for further medicinal chemistry efforts. We hope this report will prime efforts toward testing the hypothesis that positive modulation of CHT transport function is a relevant therapeutic mechanism in an appropriate cholinergic deficit model to determine level of restoration of function vs. positive controls such as acetylcholinesterase inhibitors.

## Author contributions

PC, EJA, CCJ, MP, MB, SEJ, YM and CLB designed, performed and analysed experiments; PC, EJA, CCJ, P, RT, SES and CLB contributed to figure generation; RT, SES contributed to compound design, selection and management; PC, EJA, SES and CLB contributed to writing of manuscript; PC, JSJ, SES, RIS and CLB contributed to critical review of manuscript; CLB was responsible for study conception and design. Other than as an employer of the relevant authors, Pfizer Ltd., did not play any role in the study design, collection, analysis and interpretation of the data.

## Funding

All funding was provided by Pfizer Ltd.

### Conflict of interest statement

All Pfizer-based authors (PC, EJA, CCJ, MP, RT, JSJ, RIS, SES, CLB) were full time employees of Pfizer Ltd., at the time of the study. MB was a full time employee of Nanion Technologies at the time of the study. SEJ generated data as part of her project as an industrial trainee at Pfizer Ltd. YM was a full time employee of Kissei Pharmaceutical Co. Ltd., and participating in a secondment at Pfizer Ltd., at the time of the study.

## Supplementary material

Supplementary Figure 1 **[^3^H] Choline uptake in the presence of 0.25% DMSO (Vehicle, blue), 1 μM HC-3 (red) and 10 μM STS (green) was monitored in untransformed SK-N-FI and SH-SY5Y cell lines alongside two clonal cell lines in the HEK293 background, CHT-WT4 and CHT-WT5, and the pool from which clones were created, CHT-WT Pool**. The inhibition by HC-3 and activation by STS in the CHT-WT4, CHT-WT5 and CHT-WT Pool cell lines was significant (Vehicle vs. HC3 and Vehicle vs. STS, *p* < 0.05 1-way ANOVA. *N* = 4). CHT-WT4 exhibited the largest % inhibition by HC-3 (~80%) compared to CHT-WT5 (~61%) and CHT-WT Pool (~69%). The fold-activation by STS was lower in CHT-WT4 compared to others suggesting that the uptake window was not saturated. No effect of HC-3 or STS was seen in SK-N-FI and SH-SY5Y lines.

Supplementary Figure 2 **Quantitative PCR used to measure expression of indicated genes in recombinant CHT cell lines (red) in HEK293 (A–C)** or SH-SY5Y **(D–F)** backgrounds compared to the appropriate parental cell line (blue). *RPL19A* is used as the housekeeping gene. A significant difference in CHT (*SLC5A7*) expression was observed between the recombinant and parental lines (*p* < 0.05, unpaired *t*-test, *N* = 3). Expression of no other gene was significantly altered. Individual data points, together with their mean, are shown.

Supplementary Figure 3 **Membranes from CHT-WT4 were treated with 100 μM Choline at pH 8.2**. The current amplitude signal measured on the SURFE^2^R platform was observed to be stable over time with no significant differences between any condition (1-way ANOVA, *N* = 4). Bars represent ± SD for each data point.

Supplementary Figure 4 **(A)** Measurement of [^3^H] Choline uptake in the presence of increasing concentration of [^3^H] Choline (*N* = 2). **(B)** 10-point dose response curves for STS generated at different time-points post addition of [3H] Choline. (*N* = 2). Bars represent ± SD for each data point. Cpm, Counts per minute; refers to uptake of [^3^H] Choline.

Supplementary Figure 5 **Selected images from Blaze results with purported CHT modulator seed molecules (PAM MKC-351 and NAM ML-352) (green) shown on the left and output molecules 1-5 shown on the right (gray)**. Fields are shown with positive (red), negative (cyan), van der Waals (yellow), and hydrophobic (orange) regions.

Supplementary Figure 6 **Table summarizing Z' for screening with [D9] Choline uptake**. ZPE (zero percent effect) represents vehicle (0.1% DMSO) and HPE (hundred percent effect) was generated with 10 μM Staurosporine.

Supplementary Figure 7 **(A)** Representative images showing artifacts induced by fixation of cells with 4% PFA for 15 min. Immunocytochemistry was performed on HEK293-CHT cells expressing a CHT construct that is tagged with the FLAG tag at the N-terminus and a V5 epitope at the C-terminal which projects intracellularly. Immunocytochemistry in fixed cells under non-permeabilized conditions results in detection of the intracellular V5 epitope (green) indicating permeabilization introduced by the process of fixing alone. Nuclei are counterstained with DAPI (blue). Images are captured at 10x magnification. **(B)** Quantification of cell surface expressed CHT by the Cellomics software in recombinant HEK293 cell lines stably expressing LV-AA (blue) or WT (red) CHT. Increase in cell surface CHT is seen in the LV-AA condition (*p* < 0.0001, unpaired *t*-test. *N* = 12). Bars represent ± SD.
