# Supplementary figures and images for: Discovery of Compounds that Positively Modulate the High Affinity Choline Transporter
Source: Front Mol Neurosci. 2017 Feb 27;10:40. doi: 10.3389/fnmol.2017.00040 (PMC5326799; doi:10.3389/fnmol.2017.00040)

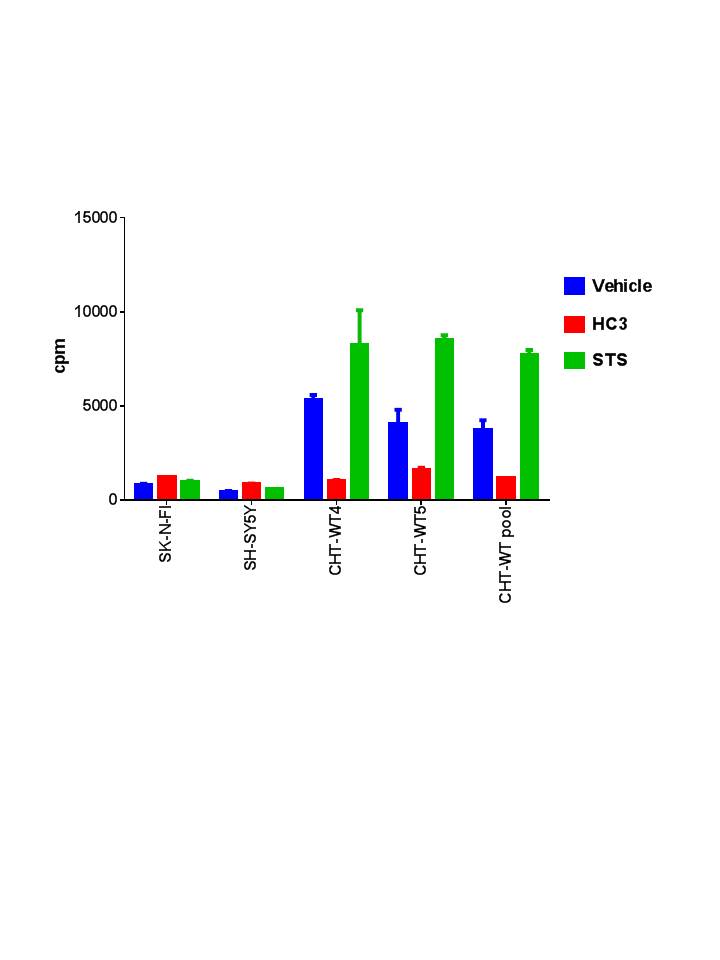

Supplement: Supplementary Figure 1 — [3H] Choline uptake in the presence of 0.25% DMSO (Vehicle, blue), 1 μM HC-3 (red) and 10 μM STS (green) was monitored in untransformed SK-N-FI and SH-SY5Y cell lines alongside two clonal cell lines in the HEK293 background, CHT-WT4 and CHT-WT5, and the pool from which clones were created, CHT-WT Pool. The inhibition by HC-3 and activation by STS in the CHT-WT4, CHT-WT5 and CHT-WT Pool cell lines was significant (Vehicle vs. HC3 and Vehicle vs. STS, p < 0.05 1-way ANOVA. N = 4). CHT-WT4 exhibited the largest % inhibition by HC-3 (~80%) compared to CHT-WT5 (~61%) and CHT-WT Pool (~69%). The fold-activation by STS was lower in CHT-WT4 compared to others suggesting that the uptake window was not saturated. No effect of HC-3 or STS was seen in SK-N-FI and SH-SY5Y lines. [file Image1.JPEG]

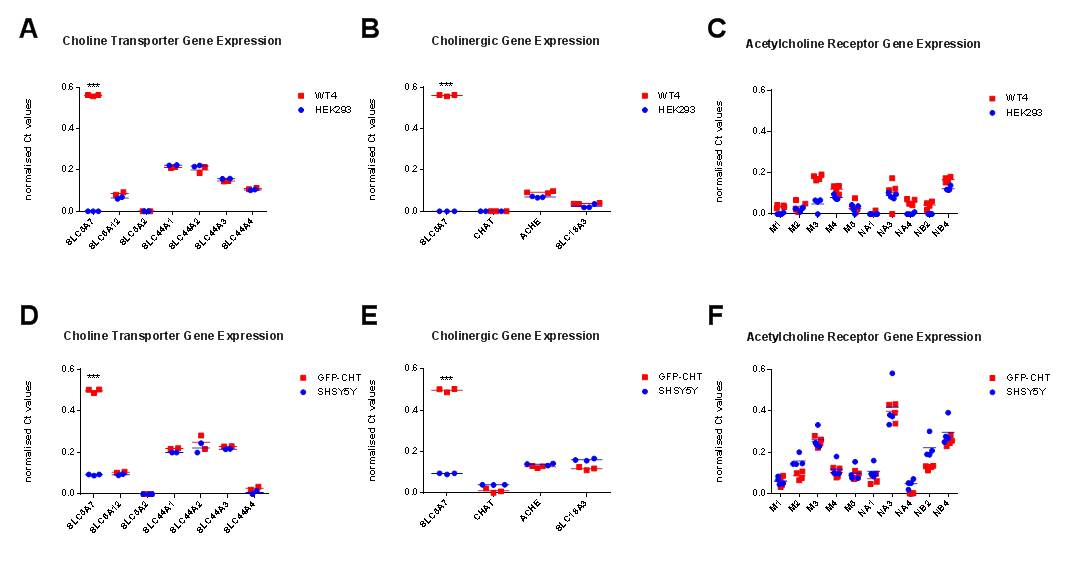

Supplement: Supplementary Figure 2 — Quantitative PCR used to measure expression of indicated genes in recombinant CHT cell lines (red) in HEK293 (A–C) or SH-SY5Y (D–F) backgrounds compared to the appropriate parental cell line (blue). RPL19A is used as the housekeeping gene. A significant difference in CHT (SLC5A7) expression was observed between the recombinant and parental lines (p < 0.05, unpaired t-test, N = 3). Expression of no other gene was significantly altered. Individual data points, together with their mean, are shown. [file Image2.JPEG]

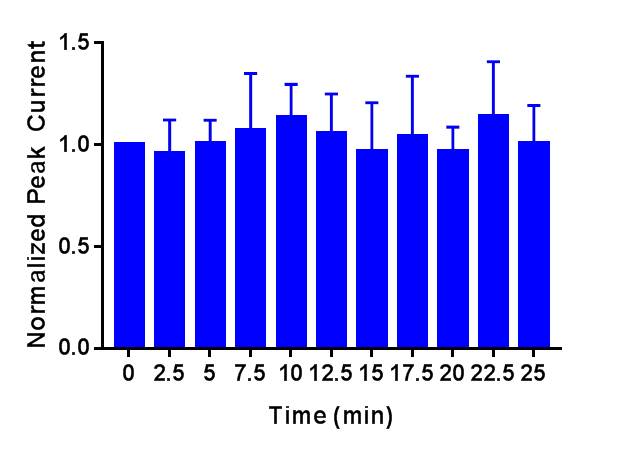

Supplement: Supplementary Figure 3 — Membranes from CHT-WT4 were treated with 100 μM Choline at pH 8.2. The current amplitude signal measured on the SURFE2R platform was observed to be stable over time with no significant differences between any condition (1-way ANOVA, N = 4). Bars represent ± SD for each data point. [file Image3.JPEG]

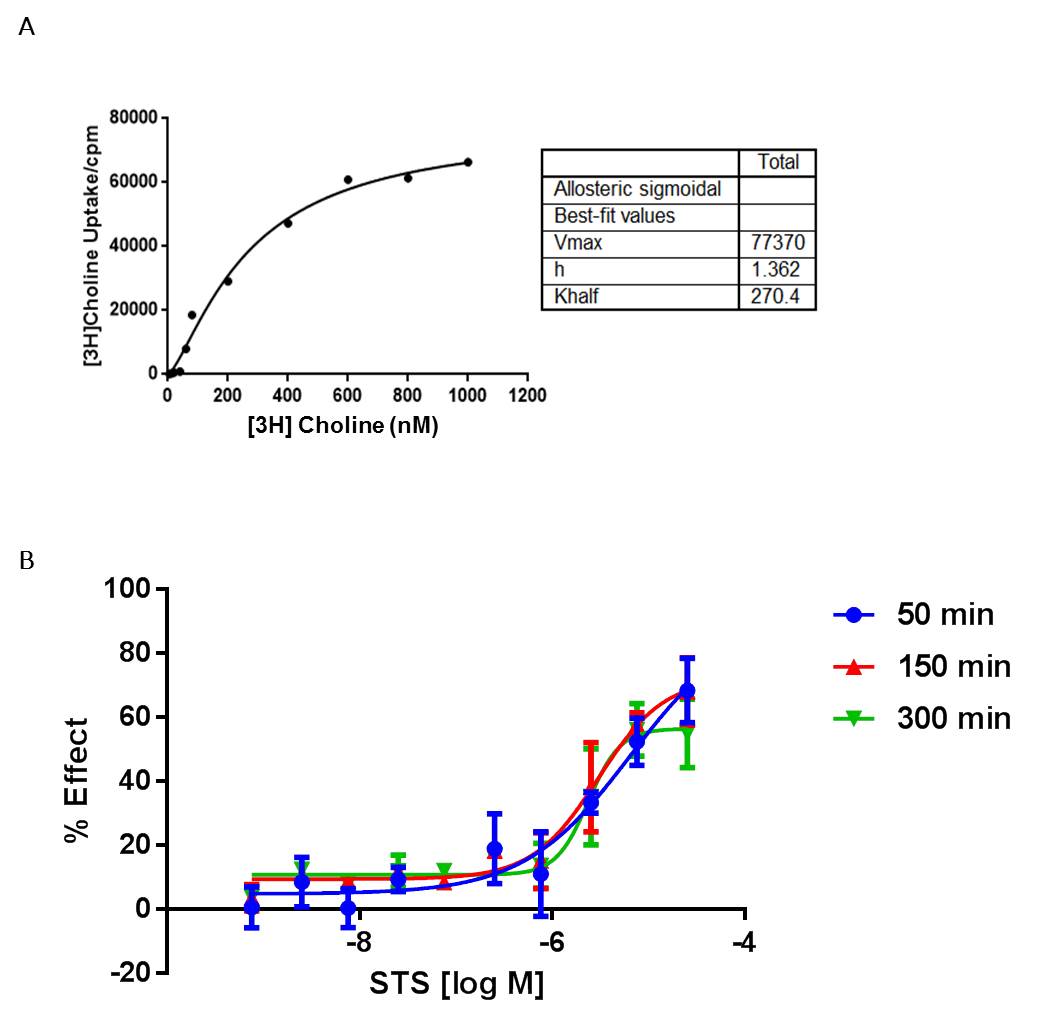

Supplement: Supplementary Figure 4 — (A) Measurement of [3H] Choline uptake in the presence of increasing concentration of [3H] Choline (N = 2). (B) 10-point dose response curves for STS generated at different time-points post addition of [3H] Choline. (N = 2). Bars represent ± SD for each data point. Cpm, Counts per minute; refers to uptake of [3H] Choline. [file Image4.JPEG]

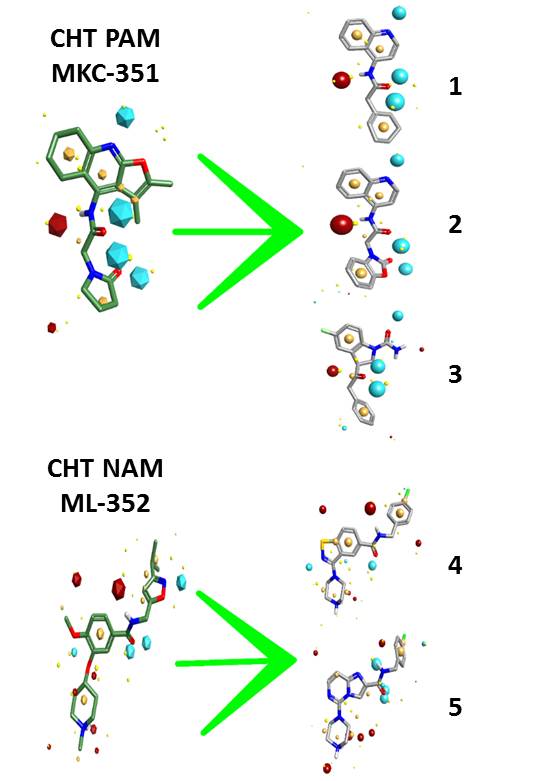

Supplement: Supplementary Figure 5 — Selected images from Blaze results with purported CHT modulator seed molecules (PAM MKC-351 and NAM ML-352) (green) shown on the left and output molecules 1-5 shown on the right (gray). Fields are shown with positive (red), negative (cyan), van der Waals (yellow), and hydrophobic (orange) regions. [file Image5.JPEG]

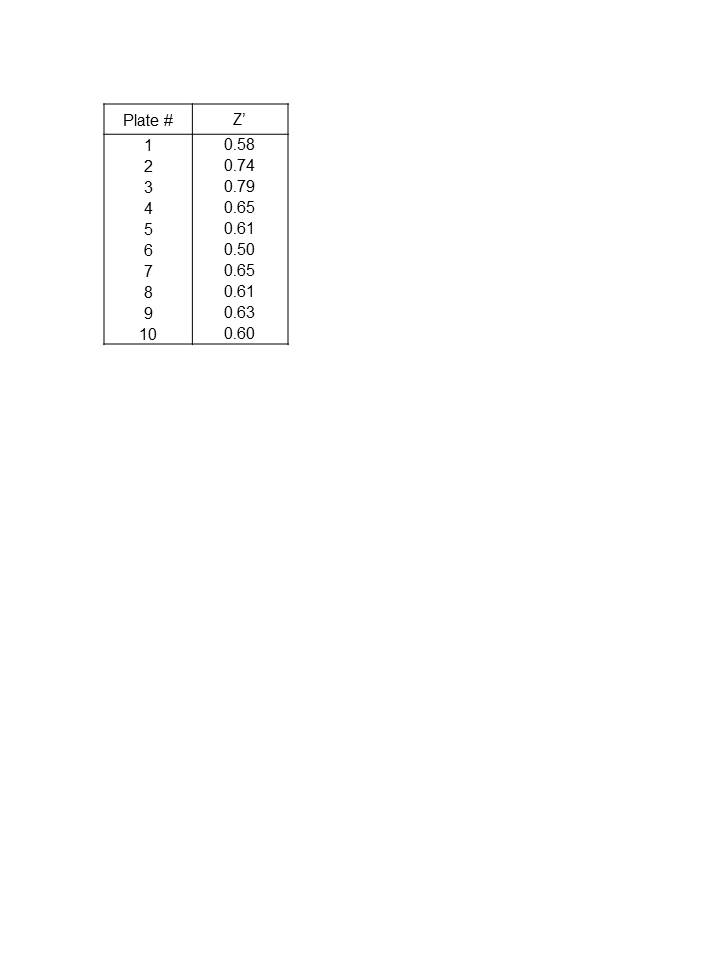

Supplement: Supplementary Figure 6 — Table summarizing Z' for screening with [D9] Choline uptake. ZPE (zero percent effect) represents vehicle (0.1% DMSO) and HPE (hundred percent effect) was generated with 10 μM Staurosporine. [file Image6.JPEG]

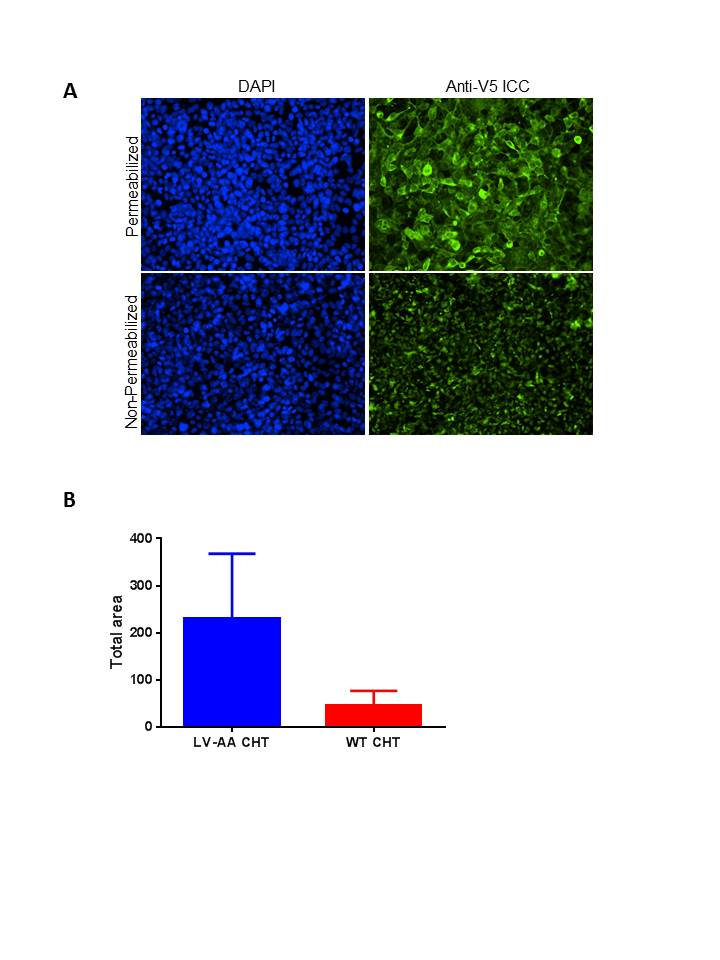

Supplement: Supplementary Figure 7 — (A) Representative images showing artifacts induced by fixation of cells with 4% PFA for 15 min. Immunocytochemistry was performed on HEK293-CHT cells expressing a CHT construct that is tagged with the FLAG tag at the N-terminus and a V5 epitope at the C-terminal which projects intracellularly. Immunocytochemistry in fixed cells under non-permeabilized conditions results in detection of the intracellular V5 epitope (green) indicating permeabilization introduced by the process of fixing alone. Nuclei are counterstained with DAPI (blue). Images are captured at 10x magnification. (B) Quantification of cell surface expressed CHT by the Cellomics software in recombinant HEK293 cell lines stably expressing LV-AA (blue) or WT (red) CHT. Increase in cell surface CHT is seen in the LV-AA condition (p < 0.0001, unpaired t-test. N = 12). Bars represent ± SD. [file Image7.JPEG]
